# Sirolimus-coated Eustachian tube balloon dilatation for treating Eustachian tube dysfunction in a rat model

**DOI:** 10.1038/s41598-024-58869-z

**Published:** 2024-04-16

**Authors:** Jeon Min Kang, Song Hee Kim, Dae Sung Ryu, Yubeen Park, Dong-Sung Won, Ji Won Kim, Jun-Kyu Park, Hong Ju Park, Jung-Hoon Park

**Affiliations:** 1 Biomedical Engineering Research Center, Asan Institute for Life Sciences, Asan Medical Center, 88 Olympic-Ro 43-Gil, Songpa-Gu, Seoul, 05505 Republic of Korea; 2 Department of Otorhinolaryngology-Head and Neck Surgery, Asan Medical Center, University of Ulsan College of Medicine, 88 Olympic-Ro 43-Gil, Songpa-Gu, Seoul, 05505 Republic of Korea; 3 Department of Research and Development, JLinker Inc., 43-22, Nanosandan 5-Ro, Nam-Myeon, Jangseong, 57248 Republic of Korea; 4 Department of Convergence Medicine, Asan Medical Center, University of Ulsan College of Medicine, 88, Olympic-Ro 43-Gil, Songpa-Gu, Seoul, 05505 Republic of Korea

**Keywords:** Drug delivery, Preclinical research, Translational research

## Abstract

Eustachian tube balloon dilatation (ETBD) has shown promising results in the treatment of ET dysfunction (ETD); however, recurrent symptoms after ETBD frequently occur in patients with refractory ETD. The excessive pressure of balloon catheter during ETBD may induce the tissue hyperplasia and fibrotic changes around the injured mucosa. Sirolimus (SRL), an antiproliferative agent, inhibits tissue proliferation. An SRL-coated balloon catheter was fabricated using an ultrasonic spray coating technique with a coating solution composed of SRL, purified shellac, and vitamin E. This study aimed to investigate effectiveness of ETBD with a SRL-coated balloon catheter to prevent tissue proliferation in the rat ET after ETBD. In 21 Sprague–Dawley rats, the left ET was randomly divided into the control (drug-free ETBD; n = 9) and the SRL (n = 9) groups. All rats were sacrificed for histological examination immediately after and at 1 and 4 weeks after ETBD. Three rats were used to represent the normal ET. The SRL-coated ETBD significantly suppressed tissue proliferation caused by mechanical injuries compared with the control group. ETBD with SRL-coated balloon catheter was effective and safe to maintain ET luminal patency without tissue proliferation at the site of mechanical injuries for 4 weeks in a rat ET model.

## Introduction

The Eustachian tube (ET) is a connector of the middle ear to the nasopharynx and has important functions in the middle ear such as ear ventilation, transport and secretion of pathogens, and protection from nasopharyngeal reflux^1^. The opening and closing of the ET are normally controlled by the movement of surrounding paratubal muscles including the tensor tympani, tensor and levator veli palatini, and salpingopharyngeus muscles; however, when the cartilaginous portion of the ET does not open properly, it causes obstructive ET dysfunction (ETD), which can lead to otitis media and cholesteatoma^2,3^. Although various pharmacologic agents, such as steroids, have been used in an attempt to relieve symptoms of ETD, they had limited effect^3^. Therefore, more direct therapeutic strategies, including laser Eustachian tuboplasty, ventilation tube insertion, and microdebrider tuboplasty, have been proposed for treating ETD^4^. However, these surgical procedures are associated with a risk of recurrence, and often result in adverse events such as ET obstruction, infection, and permanent perforation of the tympanic membrane^5^.

Eustachian tube balloon dilatation (ETBD) is emerging as a preferred alternative therapeutic option for obstructive ETD and has shown promising results^6–10^. Balloon dilatation in the cartilaginous portion of the ET can suppress adhesion and enlarge the mucous membranes; symptoms related with ETD are significantly improved in more than 70% of patients treated with ETBD^11^. However, resistant cases of ETD, such as patients who do not respond to primary ETBD, require repeated ETBD. These have a major negative impact on the quality of life of patients and are associated with recurrent symptoms^12^. Therapeutic mechanisms of ETBD have not yet been fully established; however, previous studies have indicated that ETBD leads to mild fractures within the ET cartilage and persistent dilation of the lumen^13^. A previous study of serial histological changes after ETBD in a rat model has reported that fibrosis of cartilage defects and increased thickness of the submucosa owing to tissue hyperplasia were observed. Although the lumen had been enlarged, luminal narrowing gradually developed over time owing to fibrotic tissue changes at the cartilage and submucosa injured by ETBD^14^.

In earlier studies, a drug-coated balloon catheter was used for homogenous delivery of anti-proliferative drugs at the target lesion through an inflated balloon to prolong patency and prevent restenosis^15,16^. Drug-coated ETBD may facilitate the compression of hypertrophic mucosal and submucosal injuries, potentially enabling healing with thinner, healthier layers and promoting rapid recovery after balloon dilation. A cobalt–chrome alloy stent eluting sirolimus (SRL), an anti-proliferative agent, effectively suppressed stent-induced tissue hyperplasia in a porcine ET model^13,14,18^. We hypothesized that SRL-coated balloon catheters would effectively prolong patency of the ET while inhibiting proliferation and/or fibrotic changes in submucosa resulting from mechanical injury caused by balloon dilation. This study aimed to investigate the effects of ETBD with SRL-coated balloon catheter to prevent tissue proliferation caused by fibrotic changes by ETBD in a rat ET model.

## Results

### Characterization of the sirolimus-coated balloon catheter

**Figure 1:**
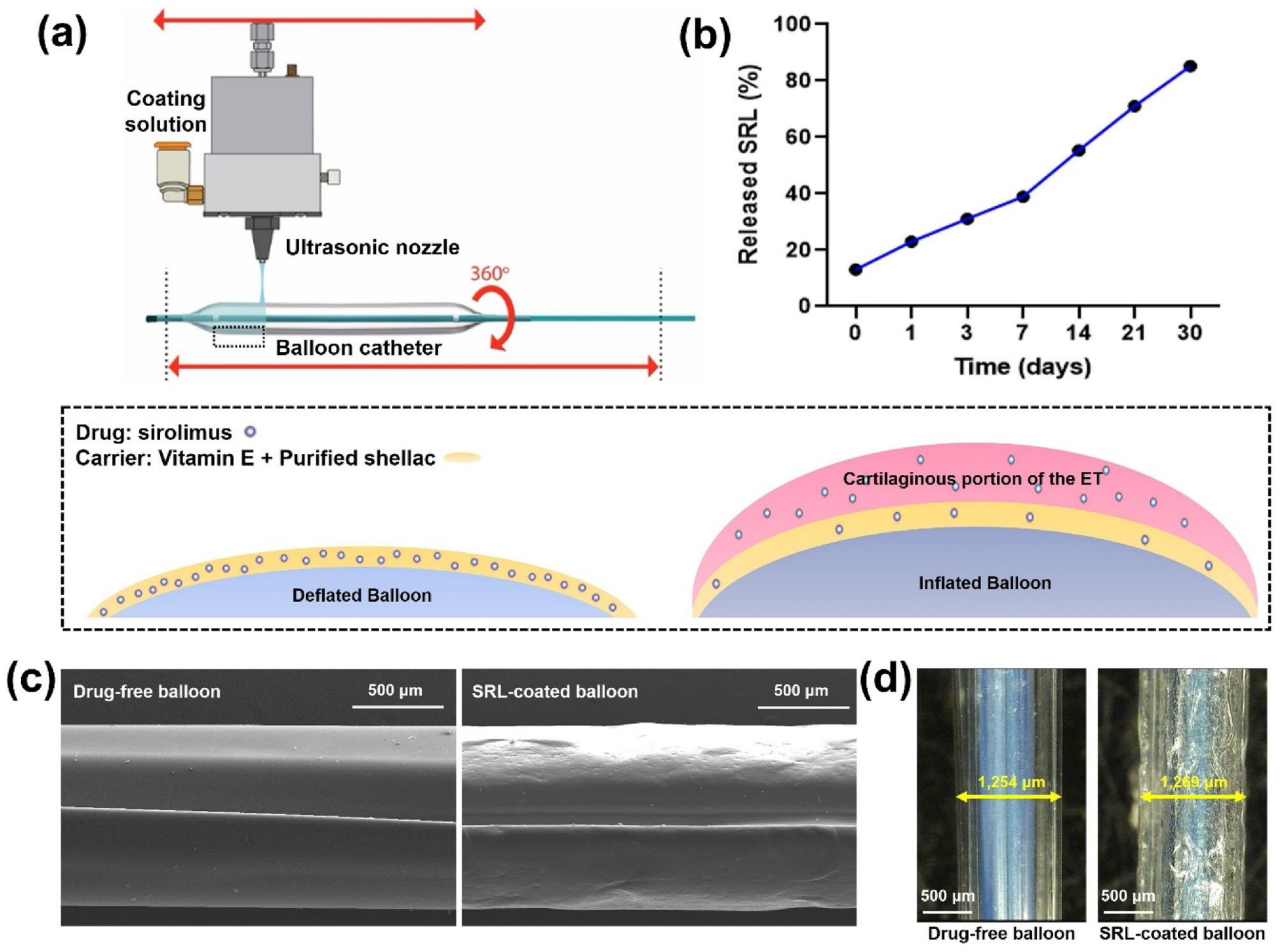
Schematic of sirolimus (SRL)-coated balloon catheter. (**a**) Schematic of SRL coating onto the balloon surface. (**b**) Graph shows the in vitro release profiles of the SRL from the balloon catheter. (**c**) Representative SEM images show the morphological characterization of the drug-free and SRL-coated balloon surfaces. (**d**) Optical images of drug-free and SRL-coated balloon surfaces were analyzed using ImageJ (U.S. National Institutes of Health, Bethesda, MD) to measure the coating thickness.

The SRL-coated balloon catheter was successfully fabricated using an ultrasonic spray coating process. The surface morphology of the SRL-coated balloon was observed to be smooth and did not have any trace of SRL crystallization (Fig. 1). The drug content and its uniformity in the SRL-coated balloon was verified using UV–Vis spectrophotometry over a period of 30 days. The coating layer was approximately 7.5-µm thick according to the optical microscopic images. (Fig. 1d). The payload of SLR was 1.27 µg/mm^2^ per balloon catheter. As shown in Fig. 1e, the SRL was slowly released from the balloon surface. The burst release at 7 days was approximately 39.5%, and approximately 85 ± 6% of the SRL was released at 30 days (Fig. 1b). SRL was securely attached to the surface of the balloon catheter.

### Technical outcomes of ETBD

**Figure 2:**
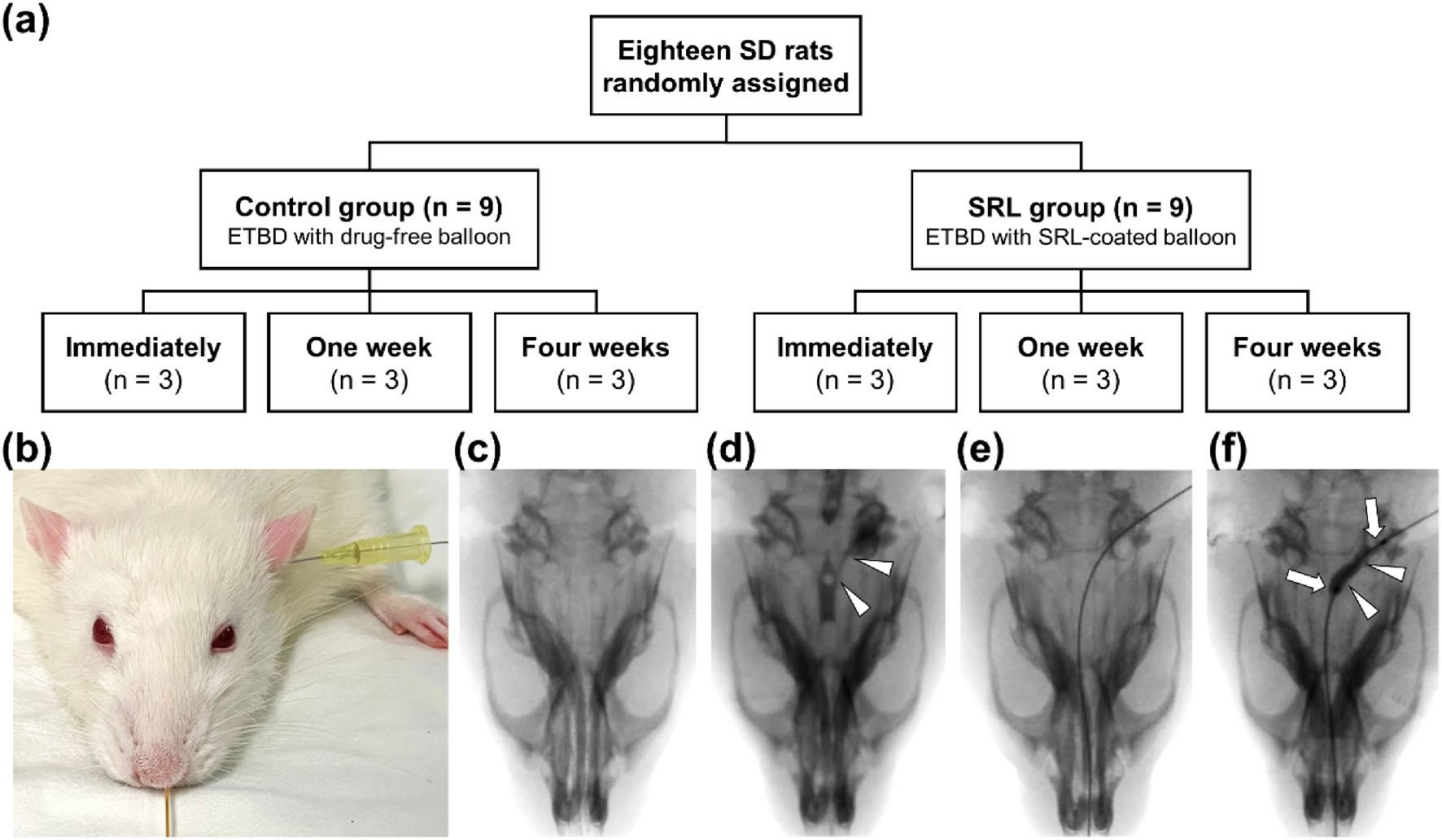
Study design and technical steps of Eustachian tube balloon dilatation (ETBD) in a rat model. (**a**) Flowchart shows the randomization of process and study follow-up. (**b**) The rat was placed in the prone position and a 24 G angiocatheter with a 0.014-inch micro guidewire was inserted from the tympanic membrane to the nasal cavity. (**c**) ETBD was performed under fluoroscopic guidance. (**d**) Contrast medium was injected through the angiocatheter to confirm the location of the rat ET (arrowheads). (**e**) Micro guidewire was inserted through the angiocatheter until it came out of the nostrils. (**f**) Balloon catheter was retrogradely advanced over the guidewire into the rat ET (arrowheads) and was inflated (arrows) to 9 atm for 1 min.

The ETBD was technically successful in all rats. Mild nosebleed was observed immediately after the procedure in two rats of the control group but resolved spontaneously within 30 min. The tympanic membrane was successfully punctured using a 24 G angiocatheter with a needle. During a contrast study of the rat ET, aspiration did not occur. A micro guidewire negotiated out of the nostrils. The balloon catheter was retrogradely advanced without any resistance. All rats survived until their respective follow-up periods without procedure-related complications (Fig. 2).

### Luminal patency of the ET after balloon dilatation

**Figure 3:**
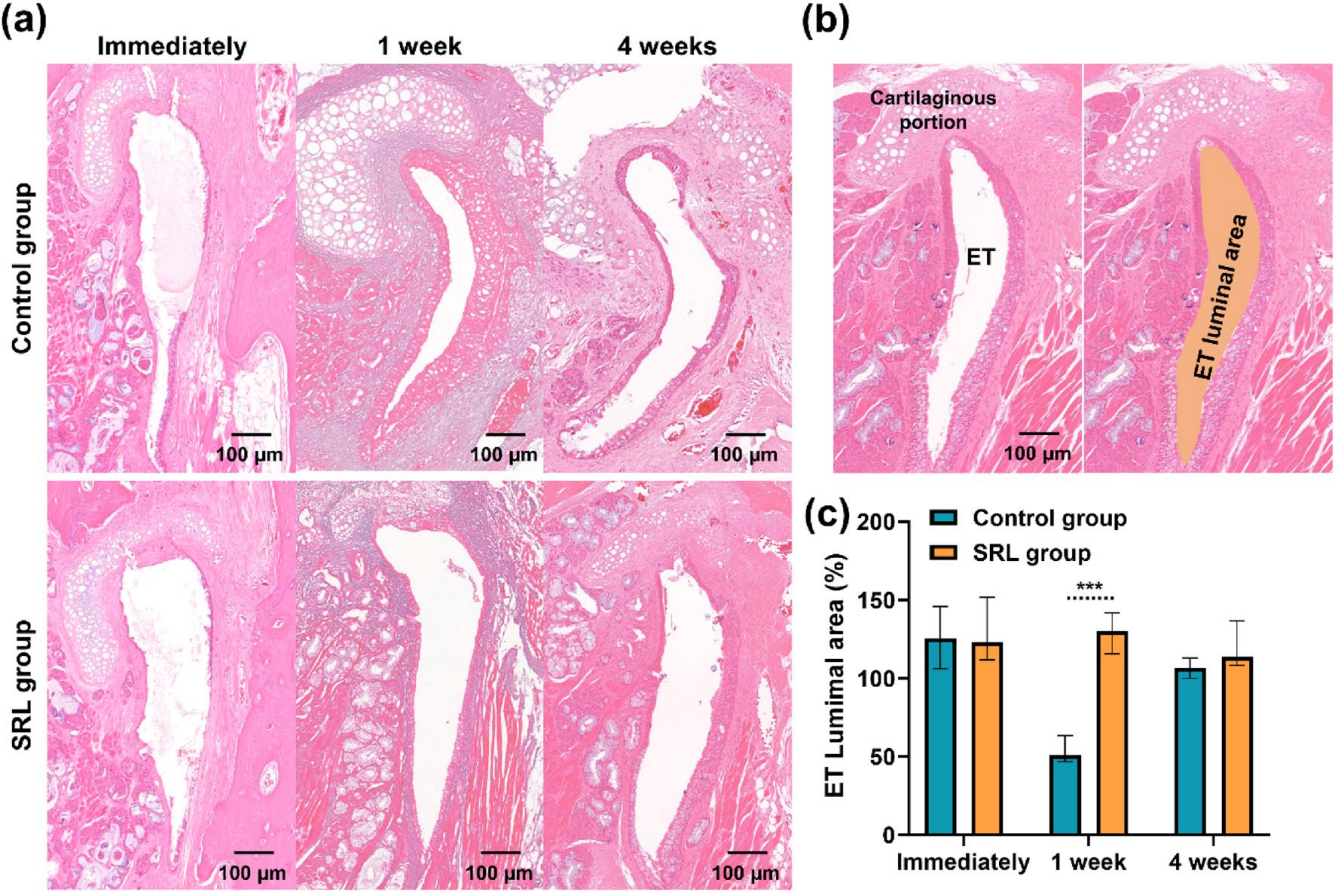
Representative histological images of the ET treated with ETBD. (**a**) The changes in ET luminal area after ETBD with or without SRL-coated balloon over time in a rat ET model. The luminal area in the control group decreased owing to proliferative tissue caused by balloon dilation; however, the luminal area in the SRL group was adequately preserved. (**b**) Histological image of the normal ET and method of analysis of ET luminal area (**c**) The graph shows the percentage of ET luminal area in the study groups. ***p < 0.001.

The luminal patency of the ET after ETBD is shown in Fig. 3. The luminal patency of the ET in both groups was relatively expanded immediately after ETBD compared with that of normal ET. However, the epithelial layers were seriously damaged in both groups. At 1 week after ETBD, severe proliferative epithelial layer and submucosa were observed in the control group. The median percentage of ET luminal area in the control group was significantly lower than that in the SRL group (50.74%, interquartile range (IQR) 47.93–58.25% vs. 130.11%, IQR 119.50–140.38%, *p* < 0.01). However, no significant differences were observed between the two groups (106.83%, IQR 101.51–111.64% vs. 113.71%, IQR 109.57–131.06, *p* = 0.065) at 4-weeks follow-up, although it was relatively large in the SRL group.

#### Histological findings

**Figure 4:**
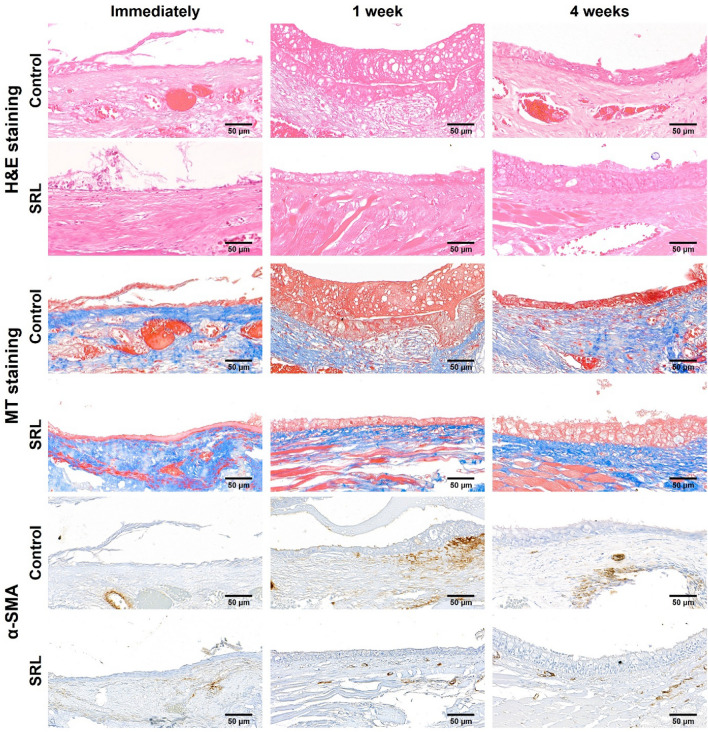
Representative histological and immunohistochemical images of hematoxylin and eosin-stained and Masson's trichrome-stained tissue, and α-smooth muscle actin at 20 × magnification.

**Figure 5:**
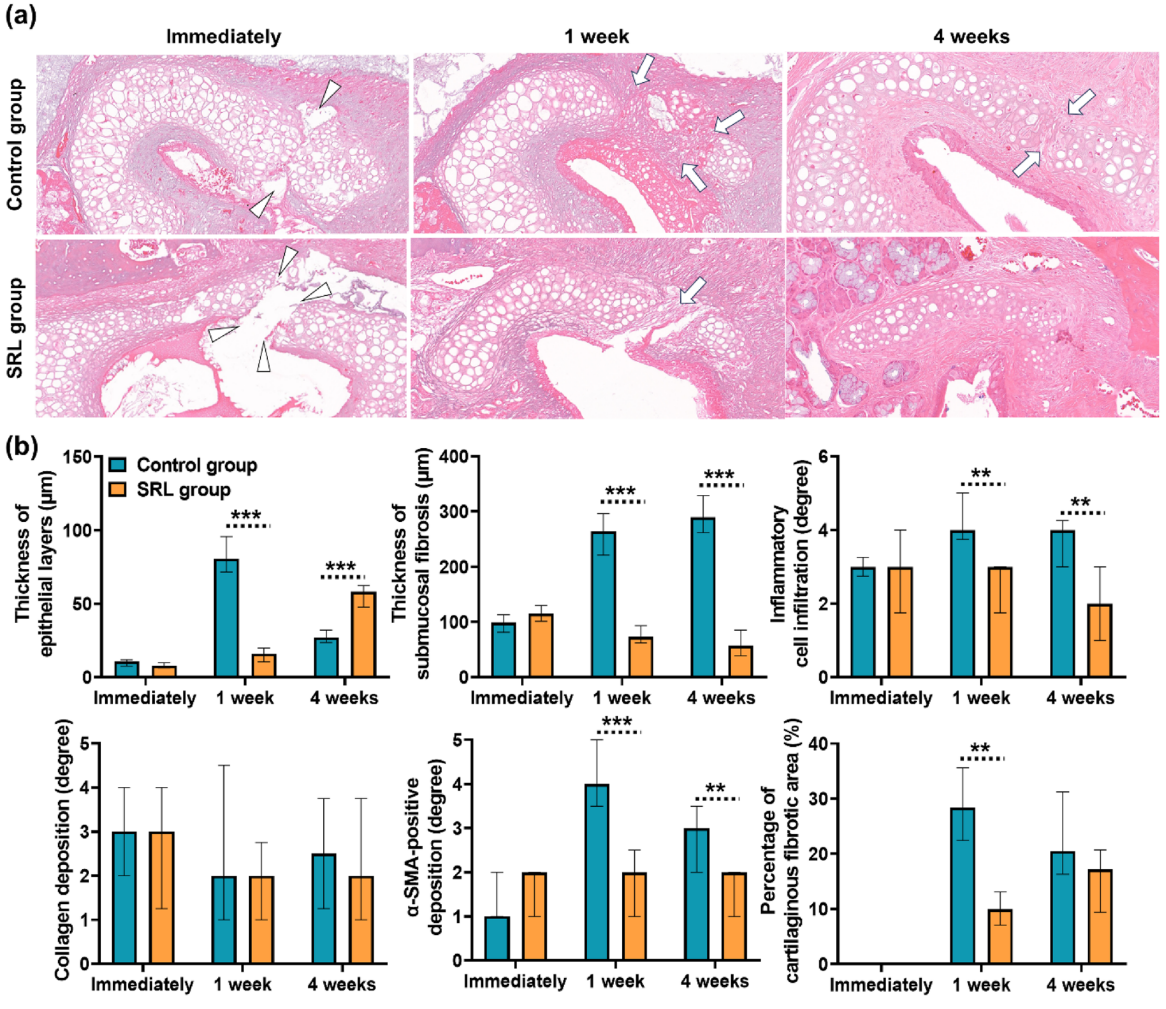
Fibrotic changes in the cartilaginous portion of the ET after drug-free or SRL-coated ETBD. (**a**) Histological images showing the ETBD-induced laceration (arrowheads) in the cartilaginous portion of the ET and the fibrotic tissue changes (arrows) that replaced the damaged areas of the cartilaginous portion. (**b**) Graphs showing the differences in histological findings between the two study groups. **p < 0.05, ***p < 0.001.

The histological and immunohistochemical findings of serial changes in the ET tissue over time are summarized in Table 1 and shown in Figs. 4 and 5. Histological evaluations performed immediately after ETBD were not significantly different between the two groups (all variables; *p* > 0.05). The median thickness of epithelial layers was significantly higher in the control group than that in the SRL group at 1 week after ETBD (80.70 µm, IQR 72.45–94.35 µm vs. 15.90 µm, IQR 10.75–18.60 µm, *p* < 0.001). However, at 4 weeks after ETBD, the median thickness of the epithelial layers in the control group was significantly lower than that in the SRL group (27.10 µm, IQR 23.85–32.05 µm vs. 58.20 µm, IQR 48.00–62.40 µm, *p* < 0.001). The thickness of the submucosal fibrosis in the control group was significantly higher than that in the SRL group after 1 week and 4 weeks (both *p* < 0.001). The degree of inflammatory cell infiltration in the control group was also higher than that in the SRL group at both 1 week and 4 weeks (*p* < 0.05, respectively). However, no significant difference was observed in the collagen deposition between the two groups over time (1 week after ETBD: 2.00, IQR 1.00–3.50 in the control group vs. 2.00, IQR 1.00–2.25 in the SRL group, *p* = 0.632; 4 weeks after ETBD: 2.50, IQR 1.75–3.25 in the control group vs. 2.00, IQR 1.00–3.25 in the SRL group, *p* = 0.737). The degree of α-smooth muscle actin (α-SMA)-positive deposition was significantly higher in the control group than in the SRL group at 1 and 4 weeks (1 week after ETBD: 4.00, IQR 4.00–5.00 in the control group vs. 2.00, IQR 1.00–2.00 in the SRL group, *p* < 0.001; 4 weeks after ETBD: 3.00, IQR 2.00–3.00 in the control group vs. 2.00, IQR 1.00–2.00 in the SRL group, *p* < 0.05).

**Table 1:** Comparison of serial histological changes over time between the study groups after Eustachian tube balloon dilatation.

	Immediately after			1-week follow-up			4-week follow-up		
	Control	SRL	*p-value	Control	SRL	*p-value	Control	SRL	*p-value
ET luminal area (%)	125.37 (108.10–144.60)	122.96 (115.01–142.71)	0.757	50.74 (47.93–58.25)	130.11 (119.50–140.38)	< 0.001	106.83 (101.51–111.64)	113.71 (109.57–131.06)	0.065
Thickness of epithelial layers (µm)	10.80 (7.75–11.90)	7.90 (6.80–9.80)	0.117	80.70 (72.45–94.35)	15.90 (10.75–18.60)	< 0.001	27.10 (23.85–32.05)	58.20 (48.00–62.40)	< 0.001
Thickness of submucosal fibrosis (µm)	99.45 (86.90–108.15)	115.30 (102.60–125.61)	0.065	264.50 (234.55–287.80)	72.80 (66.30–88.80)	< 0.001	290.05 (262.60–324.00)	57.15 (45.20–83.60)	< 0.001
Inflammatory cell infiltration (degree)	3.00 (3.00–3.00)	3.00 (2.25–3.75)	0.765	4.00 (4.00–4.75)	3.00 (2.25–3.00)	0.005	4.00 (3.25–4.00)	2.00 (1.25–2.75)	0.003
Collagen deposition (degree)	3.00 (2.00–4.00)	3.00 (1.75–4.00)	0.707	2.00 (1.00–3.50)	2.00 (1.00–2.25)	0.632	2.50 (1.75–3.25)	2.00 (1.00–3.25)	0.737
α-SMA (degree)	1.00 (1.00–2.00)	2.00 (1.00–2.00)	0.747	4.00 (4.00–5.00)	2.00 (1.00–2.00)	< 0.001	3.00 (2.00–3.00)	2.00 (1.00–2.00)	0.044
Fibrotic area at the cartilaginous portion of the ET (%)	–	–	–	28.40 (24.88–33.20)	10.00 (8.15–11.78)	0.001	20.50 (17.55–28.03)	17.15 (11.03–20.35)	0.102

Data are presented as median (interquartile range). *Bonferroni-corrected Mann–Whitney U test.

### Cartilaginous fibrosis of the ET after balloon dilatation

The cartilage fractures of the cartilaginous portion of the ET were observed in 14 (77.7%) of 18 rat ETs (Fig. 5a). The cartilaginous defects were filled with fibrosis over time. The percentage of fibrotic area at the cartilaginous portion of the ET in the control group was significantly higher than that in the SRL group at 1-week follow-up (*p* < 0.001). However, no significant difference was observed between the two groups at 4-week follow-up, although the percentage in the control group was relatively high than that in the SRL group (20.50%, IQR 17.55–28.03% vs. 17.15%, IQR 11.03–20.35%, *p* = 0.102).

## Discussion

The SRL-coated balloon catheter was successfully fabricated using an ultrasonic spray coating technique with a coating solution composed of SRL, purified shellac, and vitamin E. The shellac is a natural glue and clear coating material. The surface of the balloon can be rendered adhesive by the shellac and the purified shellac may increase attachment of the SRL to balloon surface and prevent SRL loss from the coating surface before the procedure. Vitamin E can reduce the inflammatory response immediately after ETBD^17^. Our in vivo study demonstrated that the degree of inflammatory cell infiltration in the SRL group was significantly lower than that in the control group. Using ultrasonic spray coating can decrease initial burst release and ensure uniform coating layers. Our in vitro results revealed that approximately 76% of the SRL was maintained on the balloon surface at 3 days.

The balloon dilatation of the cartilaginous portion of ET has been considered a safe and effective procedure for treating obstructive ETD^18^. The precise mechanism underlying tissue injuries owing to balloon dilation in the ET remains unclear and may involve a combination of factors, including direct pressure, hypoxia/hypoperfusion, and shearing trauma^13,19,20^. When BD is performed, the luminal area is significantly expanded while simultaneously causing crush injury to the epithelium and submucosa^21^. ETBD failure may be related to excessive tissue hyperplasia with fibrotic tissue formation caused by crush injury. Recurrence rates of 10–46% have been reported within the first month after ETBD^11,22,23^. Our histological findings revealed a significant increase in submucosal fibrosis over time in the rat ET treated with ETBD. However, the thickness of the submucosal fibrosis in the SRL group was significantly lower than that in the control group. The luminal patency of the rat ET treated with SRL-coated ETBD was maintained for 4 weeks.

SRL interrupts the mammalian target of rapamycin (mTOR) intracellular signaling pathway and inhibits phases of proliferation by blocking cell cycle progression at the G1/S transition^24–27^. Thus, SRL acts in the initial phase of the cell cycle and is considered to have lower toxicity compared with drugs that act in later stages of the cell cycle^28^. The severe proliferative epithelial layer was observed in the control group at 1 week after ETBD. In contrast, the SRL group had a significantly lower regenerated thickness of the epithelial layers. At 4 weeks after ETBD, the epithelial layers in the SRL group were increased compared with the control group. SRL inhibits the initial tissue hyperplasia owing to mechanical injuries and has low toxicity, suggesting that it does not affect the normal regeneration of epithelial layers after ETBD. The proliferative phase begins from days 4–14 of the wound healing process, with an excessive increase in fibroblasts and myofibroblasts^29^. Induction of fibrosis in the submucosa of ET increases the rigidity of the lumen. The strengthened ET lumen maintains patency^30,31^. However, excessive submucosal fibrosis not only hardens the ET lumen but may also affect ET movement and lead to patulous ETD. Richard et al. reported that the rate of patulous ETD symptoms was 7% after ETBD^32^. This rate is higher than the natural prevalence of patulous ETD^33^. α-SMA is commonly used as a marker of myofibroblasts, which mediate wound contraction but cause fibrosis when persist in the tissue^25^. The thickness of submucosal fibrosis and degree of α-SMA-positive deposition were significantly lower in the SRL group than in the control group both at 1 week and 4 weeks. SRL-coated balloons can be effective from a long-term perspective by suppressing fibrosis.

The ET cartilage is an elastic cartilage, which has the highest elasticity among cartilages^4,34,35^. Small full-thickness defects of elastic cartilage are primarily replaced by fibrocartilage, whereas partial defects have voids filled by the infiltration of fibrous scar tissue^34^. The cartilage fractures are closely related to the size and pressure of the balloon catheter. Many studies have emphasized the importance of the size and pressure of the balloon catheter used for ETBD^18,36–39^. In our study, ETBD was performed for 1 min at a pressure of 9 atm using a 1.25-mm diameter balloon catheter in the left ET of the rats, and partial cartilage fractures were observed in 77.7% of all ETs after ETBD. The fibrotic area in the cartilaginous portion increased at 1 week and decreased at 4 weeks after ETBD in the control group. In the SRL group, the fibrotic area gradually increased over time but was relatively low compared with that in the control group. This suggests that the infiltration of fibrous scar tissue into partial defects of the fractured cartilage is delayed owing to SRL but not replaced by elastic cartilage. The lack of response to repeat ETBD after the failure of primary ETBD^40^ may be owing to the increased fibrotic area of the fractured cartilage. Infiltration of fibrous scar tissue may interfere with cartilage expansion and contraction and result in additional defects^41,42^. Therefore, to prevent cartilage fractures, the size of the balloon catheter should be determined after diagnosing the patient's ET. Although SRL did not prevent fibrotic scar tissue infiltration in the cartilaginous portion, the histological results of this study provide the basis for early preclinical findings to address the limitations of ETBD.

The ETBD via trans-tympanic approach was technically successful in all rats without severe procedure-related complications. The rat ET model has several advantages compared with large animal models, including cost effectiveness, readily available, animal study on large sample size, and easy to use. Pig or sheep ET model has been commonly used, but these models are extremely expensive and is available only in a few laboratories. Although the anatomical structure is similar to that of human or large animal, the anatomy of the rat ET is relatively small, narrow and complicated^43^. We chose appropriate interventional equipment such as balloon catheter and guidewire and successfully performed ETBD in a rat ET model. Our ETBD rat model proved to be feasible for use in the rat ET and an efficient approach to stimulate balloon-induced mucosal injuries as a potential model for reproducing the mechanisms of mucosal injuries with healing process.

This study had several limitations. First, the number of animals was limited, and a robust statistical analysis could not be performed. Second, ETBD was performed in the normal rat ET and the wound healing process following ETBD may differ from that of obstructive ETD. Third, in vivo pharmacokinetic studies of the SRL-coated balloon were not conducted. Fourth, histological analysis was determined subjectively according to the distribution and density of the cells or antibody positive deposition. Finally, functional evaluation of the rat ET could not be performed. Nevertheless, although additional studies are required to identify the optimal SRL dose and verify its efficacy and safety in a large animal model, our study results support the basic concept of SRL-coated ETBD to prolong ET luminal patency in a rat model.

ETBD performed using an SRL-coated balloon catheter was effective and safe to maintain ET luminal patency without tissue proliferation at the site of mechanical injuries for 4 weeks in a rat ET model. In this study, we were able to successfully coat SRL with purified shellac and vitamin E using the ultrasonic spray coating. The SRL-coated ETBD significantly suppressed tissue proliferation compared with the control group in balloon-induced mechanical injuries. Although further studies are needed to investigate the efficacy and safety of SRL-coated ETBD for obstructive ETD, the SRL-coated ETBD should be a promising therapeutic option for patients with obstructive ETD or refractory ETD after ETBD.

## Methods

### Preparation of sirolimus-eluting balloon catheter

The balloon catheter (JLinker Inc., Jangseong, Korea) for the rat ET was 1.25 mm in diameter and 10 mm in length. SRL was coated onto the surface of the expanded balloon catheter using an ultrasonic coating machine (DEB coating machine, Noanix Inc., Korea). The coating solution consisted of vitamin E (5 mg, WonPoong Pharm. Co., Ltd, Seoul, Korea), purified shellac (5 mg, WonPoong Pharm. Co., Ltd), and SRL (5 mg, Biocon, Bangalore, India) dissolved in 5 mL of tetrahydrofuran. The balloon catheter was placed under an ultrasonic spray nozzle with a rotating shaft. The coating solution was sprayed over the balloon surface through the ultrasonic nozzle at room temperature (24 ± 1°C) with flow rate of 50 μL/min under a nitrogen atmosphere. The SRL-coated balloon catheter was vacuum-dried for 24 h. The prepared SRL-coated balloon catheter was crimped for the procedure using a crimping machine (J-Crimp; Blockwise Engineering, Tempe, Ariz).

### Morphological analysis of sirolimus-coated balloon catheter

The surface morphology of the surface of the SRL-coated balloon was analyzed using optical and scanning electron microscopy (SEM; Sigma 300, Carl Zeiss, Oberkochen, Germany). The SRL-coated sample was fixed on an aluminum pin stub mount using carbon tape, and then the surface of the samples was analyzed to ensure distribution of the coating solution over the balloon surface.

### Drug amount and release of sirolimus-coated balloon catheter

Amount of SRL on the balloon catheter was measured using a UV–Vis spectrophotometer at 278 nm. To determine the amount released of SRL from the balloon surface after inflating, a standard curve was prepared by measuring the concentration of SRL, which was dissolved in phosphate buffer saline (pH 7.4) and acetonitrile in the range of 0.5–32.5 µg/mL. The expanded balloon catheter was inserted within a silicone tube of 3 mm in diameter. The tubes were immersed in 5 mL of phosphate-buffered saline (PBS) within tinted vials and subjected to agitation at 100 rpm at 37°C. At predetermined intervals, the balloon sample was extracted from the tube, and the PBS medium was refreshed with a new solution. The concentration of released SRL was identified at immediately after coating, 1, 3, 7, 14, 21, and 30 days.

#### Animal study design

The protocol of animal study was approved by the Institutional Animal Care and Use Committee (No. 2023-40-112), and the animals used in this study were handled in accordance with the United States National Institutes of Health guidelines for the humane handling of laboratory animals. Overall, 21 male Sprague–Dawley rats, aged 12 weeks (weighing, 350–400 g; A BIO, Suwon, Korea) were used in this study. Of these, 18 rats were randomly assigned to the control and the SRL groups. The control group (n = 9) received a drug-free ETBD, whereas the SRL group (n = 9) received an SRL-coated ETBD. The rats in each group were euthanized immediately (n = 3), at 1 week and 4 weeks (both n = 3) after ETBD. The remaining three rats were euthanized to serve the normal ET as a reference without undergoing ETBD. All rats were housed under a 12-h control cycle at 55% ± 10% relative humidity and 24 ± 1°C environmental temperature. All rats were euthanized by administering inhalable pure carbon dioxide at the respective times.

### Eustachian tube balloon dilatation

For all rats, anesthesia was performed using intramuscular injection of 50 mg/kg zolazepam and tiletamine (Zoletil 50; Virbac, Carros, France) and 10 mg/kg xylazine (Rompun; Bayer Healthcare, Leverkusen, Germany). The tympanic membrane was punctured through the external auditory canal using a 24 G angiocatheter with a needle (BD Angiocath Plus; Becton Dickinson, Franklin Lakes, NJ, USA). The angiocatheter was carefully inserted into the tympanic cavity and 1 mL of contrast medium was injected to visualize the tympanic orifice of the ET under a fluoroscopic guidance (MeteoR, NanoFocusRay Co., Iksan, Korea). A 0.014-inch micro guidewire (Transend; Boston Scientific, Marlborough, MA, USA) was inserted through the angiocatheter and negotiated consequently out of the nasal cavity. After the angiocatheter was removed, the balloon catheter was retrogradely advanced over the micro guidewire into the nasal cavity and located at the ET using fluoroscopic guidance. The balloon catheter was fully inflated to 9 atm as determined by the pressure gauge monitor of the balloon inflator (Genoss, Suwon, Korea) for 1 min^14^. The micro guidewire and balloon catheter were pulled out from the ET after ETBD.

### Histological examination

Mandible and anatomical structures anterior to the soft palate were surgically resected to explore the nasopharyngeal opening of the ET after euthanasia^14^. Then, soft tissues around the ET orifice were removed. The dissected head samples were fixed in 10% neutral buffered formalin for 48 h and then decalcified for 2 weeks. The samples were embedded in a paraffin block and coronally sliced at intervals of 200 μm from the nasopharynx to the posterior wall of the tympanic cavity, which yielded three sections per sample. The slides containing the sections were stained with hematoxylin–eosin (H&E) and Masson’s Trichrome (MT). H&E-stained slices were evaluated for the percentage of ET luminal area, thickness of epithelial layers, thickness of submucosal fibrosis, degree of inflammatory cell infiltration, and percentage of fibrotic area at the cartilaginous portion of the ET^13,14^. The thickness of submucosal fibrosis was vertically measured from the tensor veli palatini muscle to the submucosal layer. The percentage of ET luminal area was calculated using the following equation: 100 × (balloon-treated ET lumen area/normal ET lumen area). The degree of inflammatory cell infiltration was classified according to the density and distribution of cells and graded as follows: 1, mild; 2, mild to moderate; 3, moderate; 4, moderate to severe; and 5, severe^23,24,29^. The percentage of fibrotic area at the cartilaginous portion of the ET was evaluated using the following formula: 100 × (fibrotic area at the cartilaginous portion/area of cartilaginous portion). On MT-stained slices, the degree of collagen deposition was subjectively classified into five grades as follows: grade 1, mild; grade 2, mild to moderate; grade 3, moderate; grade 4, moderate to severe; and grade 5, severe^23,24,29^. All stained samples were histologically visualized and measured via Caseviewer (3D HISTECH, Budapest, Hungary). This study was examined by three observers blinded to the experimental groups.

#### Immunohistochemistry

Immunohistochemistry (IHC) examination was performed on paraffin-embedded sections using α-smooth muscle actin (α-SMA; Abcam, Cambridge, England). The α-SMA-positive deposition was subjectively classified into five grades (1, mild; 2, mild to moderate; 3, moderate; 4, moderate to severe; and 5, severe)^25^.

#### Statistical analysis

Data were expressed as the median and interquartile range (IQR). Differences between groups were analyzed using a Bonferroni-corrected Mann–Whitney *U* test. Statistical analyses were performed using SPSS (version 27; IBM, Chicago, IL, USA). A *p*-value of < 0.05 was considered to indicate statistical significance.

### Ethics declarations

All experiments were performed in accordance with relevant ARRIVE guidelines and regulations.

### Approval for animal experiments

All experiments were approved by the Institutional Animal Care and Use Committee of the Asan Institute for Life Sciences (IACUC-2023-40-112).

## Data Availability

The authors confirm that the data supporting the findings of this study are available within the article. Raw data that support the findings of this study are available from the corresponding authors upon reasonable request.
